# Trends in the aetiology of urogenital fistula: a case of ‘retrogressive evolution’?

**DOI:** 10.1007/s00192-015-2919-y

**Published:** 2016-01-07

**Authors:** Paul Hilton

**Affiliations:** Newcastle University, Newcastle upon Tyne, UK

**Keywords:** Urinary fistula, Vaginal fistula, Ischaemia, Iatrogenic disease, Caesarean section, Training

## Abstract

It has long been held as conventional wisdom that urogenital fistulae in low-income and middle-income countries are almost exclusively of obstetric aetiology, related to prolonged neglected obstructed labour, whereas those seen in high-income countries are largely iatrogenic in nature. There is, however, a growing perception amongst those working in the field that an increasing proportion of urogenital fistulae in low-income and middle-income countries may be iatrogenic, resulting from caesarean section. Recent studies suggest that adverse patterns of care may also be emerging in high-income countries; an increase in the risk of both vesicovaginal and ureterovaginal fistulae following hysterectomy has been reported, concurrently with the reduction in overall use of the procedure. These apparent secular trends are discussed in the context of evolution of practice, teaching and training in obstetrics and gynaecology.

## Introduction

*Plus ça change, plus ça empire*– after J.-B.A. Karr (and ‘Voltaire’)

The term ‘retrogressive’ is defined as:

characterized by retrogression: as

**a:** declining from a better to a worse state <*retrogressive* disease>

**b:** passing from a higher to a lower level of organization <*retrogressive* evolution> [1]

More specifically, ‘retrogressive evolution’, is seen as:

A developmental trend in evolution resulting in simplification of an organism, usually through the complete or partial loss of one or more structures [2]

Charles Darwin in his book ‘*On the Origin of Species*’ in 1859 proposed the theory of natural selection to explain the progressive advance of evolutionary processes [3]. In contrast, in his 1868 work ‘*On the Origin of Genera*’, E.D. Cope proposed that evolution may proceed not only on lines of acceleration but also of retardation [4]. That is, that evolution had been not only progressive, but at times retrogressive [5]. Huxley in his collected essays entitled ‘*The Struggle for Existence in Human Society*’ in 1888 similarly emphasised: ‘It is an error to imagine that evolution signifies a constant tendency to increased perfection. … Retrogressive is as practicable as progressive metamorphosis’ [6]. What, then, can we say about the evolution of our surgical practices in relation to urogenital fistula?

It is conventional wisdom that urogenital fistulae in low-income and middle-income countries are almost exclusively of obstetric aetiology, related to prolonged neglected obstructed labour, whereas those seen in high-income countries are largely iatrogenic in nature. Furthermore, the pattern of disease reported from low-income and middle-income countries in recent years apparently reflects that seen in high-income countries over a century ago. One might anticipate that this would augur well for rapid progress towards the eradication of fistula by effective primary and secondary prevention in low-resourced environments, and the thought that retrogressive evolutionary processes might be at work in our surgical development is anathema; emerging evidence, however, suggests that some consideration of this possibility is justified.

## Accepted views on aetiology of urogenital fistula in low-income and middle-income countries

There is little high-level evidence in relation to urogenital fistula, and recent systematic reviews are based largely on small case series from individual surgeons working in single institutions (Hillary et al., submitted for publication), [7, 8], with only three randomised controlled trials (RCTs) published on any aspect of the subject [9–11]. Most published studies from low-income and middle-income countries have shown that over 95 % of fistulae are of obstetric aetiology, with over 80 % following prolonged neglected obstructed labour (Hillary et al., submitted for publication), [7]. The main pathophysiological factor is ischaemia from soft-tissue compression, the likelihood of fistula formation being compounded by several areas of potential delay – in the decision to seek care, in arrival at a health-care facility, and in the provision of adequate care (often referred to as ‘Maine’s delays’) [12]. Caesarean section and instrumental delivery were associated in 9 % and 2 % of cases, respectively, in one recent systematic review (Hillary et al., submitted for publication).

Current estimates of the global prevalence of untreated obstetric fistulae vary from 654,000 to 3,500,000 [7]. National community-based estimates of prevalence are consistent at 1.5 to 1.7 per 1,000 women surveyed in Ethiopia [13], Malawi [14] and Bangladesh [15]. Reports of the incidence of obstetric fistula show much greater variation, with a difference between estimates of approximately tenfold. The only prospective population-based study found an overall incidence of 0.10 per 1,000 births, and an incidence in rural areas of 1.24 per 1,000 births in West Africa [16]. On the basis of this work these authors estimated a minimum of 33,451 (95 % confidence interval 4,050 – 120,413) new cases annually in rural sub-Saharan Africa. Hospital-based reports show the incidence to be between 0.6 and 6.5 per 1,000 births [7].

## Recent trends in ‘obstetric’ fistula

There are no published longitudinal data on trends in incidence of obstetric fistulae, although recent data suggest that there may be a reduction in new cases seen in Ethiopia. There is, however, a growing perception that an increasing proportion of urogenital fistulae in low-income and middle-income countries are iatrogenic in aetiology, rather than being true ‘obstetric’ fistulae from ischaemic mechanisms. Although this impression relates primarily to obstetric interventions, especially caesarean section, iatrogenic fistulae are also seen increasingly following gynaecological procedures in some areas.

A study of 987 patients managed in the Hamlin Fistula Ethiopia (HFE) hospital at Barhirdar between 2004 and 2007 found 356 (36 %) to be type 1 on the Goh classification (i.e. more than 3.5 cm from the external urinary meatus) [17]. In a further study of fistulae managed in three other of the six HFE hospitals, Wright and colleagues found the proportion of fistula referrals classified as being ‘high’ (presumed to be of iatrogenic aetiology) increased to 36 %, albeit concurrently with a significant reduction in the annual number of referrals seen (J. Wright, personal communication of unpublished data). It should be noted, however, that the Goh classification is more appropriate for obstetric than iatrogenic fistulae [18], and neither the designation as ‘Goh type 1’ nor ‘high’ can be taken as an absolute indication of aetiology, both being likely to overstate the true proportion of iatrogenic cases.

In a study of 5,959 women managed in 65 facilities across 11 countries mainly in East Africa over an 18-year period, Raassen et al. found 805 fistulae in 788 women (13.2 % of the total) to be iatrogenic in nature [19]. Waaldijk, with a personal experience of over 25,000 conservative and surgical fistula treatments in over 20,000 women treated over three decades, reported that in 139 of his first 1,000 cases the woman had had a caesarean delivery in her index pregnancy, and 55 of the 1,000 cases (5.5 %) were probably iatrogenic in aetiology; this compared to 380 of his last 1,000 cases, with 163 of the 1,000 (16.3 %) probably iatrogenic. Hence, although the confirmation of the exact aetiology may not be wholly accurate, there is reasonable evidence of a threefold increase in the rate of iatrogenic fistulae over this period in Nigeria and Niger. Similarly, faculty surgeons from other fistula units in Nigeria, Kenya, Uganda, Sierra Leone and Pakistan report an increasing the number of post-caesarean section fistulae seen in recent years; although data are limited, such cases may now represent more than one-third of fistulae seen in some areas.

## Factors behind recent trends seen in ‘obstetric’ fistula

### Access to skilled maternity services and obstetric intervention

Although even in the 21st century there remain areas of the world where witchcraft and neo-religious practices may opine differently [20], the significance of obstructed labour as a contributory factor to maternal and perinatal mortality and morbidity is well established; the need to relieve obstruction in order to limit these consequences, including fistula formation, is well recognised. In a recent statement, the World Health Organization (WHO) emphasised that whilst caesarean section can effectively prevent maternal and perinatal mortality and morbidity, when medically justified, there is no evidence of benefit to women or infants who do not require the procedure on medical grounds [21]. Caesarean section is associated with risks that can extend beyond the current delivery and are greater in women with limited access to comprehensive obstetric care [21]. The WHO statement also emphasises the imperative to provide caesarean sections to women in need, rather than striving to achieve any specific intervention rate. In many resource-poor settings, access to skilled care and crucial interventions is limited, and the rate of caesarean delivery is a marker for the availability and use of obstetric services in these situations [22]. In a review of trends in caesarean section rate in Asia and sub-Saharan Africa, Cavallaro et al. point out the association between poverty and access to healthcare; although rates up to 22 % were seen (especially in wealthier urban populations), among the poorest quintile of the population, caesarean sections accounted for less than 2 % of deliveries in 21 of 26 countries studied [23]. Hence, limited access to obstetric intervention is likely to remain a significant contributory factor in generating fistula, particularly amongst the rural poor.

### The appropriate workforce

There is a critical workforce shortage in many areas of the world, particularly in low-income and middle-income countries. This inevitably overstretches those personnel who are present, and may contribute to patient abuse and neglect. Caesarean section is often delegated to the most junior member, often a doctor with minimal training, supervision or mentorship. Decision-making is often poor, and hence caesarean delivery may be undertaken in inappropriate cases, with inappropriate timing, and/or using inadequate surgical techniques. Preoperative risk assessment is likely to be inadequate, with failure to recognise the increased risk of damage to the urinary tract (at caesarean section or hysterectomy) associated with a previous operative delivery [24, 25]. In some areas financial incentives rather than clinical priorities may dictate the choice of mode of delivery, and indeed knowledge and experience of alternative ways of delivery, especially of a dead baby, are often lacking. In the right circumstances emergency caesarean section may be life-saving, but when performed inappropriately it may increase maternal risk whilst being too late to improve perinatal outcome [26].

### Use of non-medically qualified clinical officers

The training of non-medically qualified clinical officers (NMQCO) has been introduced into a number of low-income and middle-income countries to boost the available cadre of personnel able to provide emergency obstetric care. In 19 out of 47 sub-Saharan African countries, NMQCOs are authorised to provide obstetric care, and in five countries (Zaire, Burkina Faso, Malawi, Mozambique and Tanzania) they are permitted to carry out caesarean sections and other emergency obstetric surgery [27]. The use of NMQCOs in providing major obstetric surgery has been found to be cost-saving in Mozambique [28], with no significant difference in outcomes compared to medical officers in Tanzania [29] and Malawi [30]. In a meta-analysis of data from 16,018 women in six non-randomised controlled cohort studies, Wilson et al. also reported no difference in mortality, although they did find an increase in postoperative complications [31]. None of these studies investigated comparative outcomes for more senior doctors, nor did they include data on fistulae specifically.

### The appropriate working environment

Whilst taken for granted in high-income countries, a safe and equipped working environment is crucial to minimising risk during surgery, and is often lacking in low-income and middle-income countries. Electricity supplies for lighting and equipment are often unreliable, and running water for scrubbing and sterilisation processes may be inconsistent. Instruments are often unavailable or in need of repair, and in keeping with the workforce issue highlighted above, skilled assistance for retraction is often unavailable. Getting access to theatre may be especially problematic at night when the difficulties in finding appropriate staff and equipment are even greater. This adds to delays in preparing the theatre, and in some areas patients families may be charged with leaving the hospital to purchase drugs and materials from off-site pharmacies before an operation can begin. These are all contributors to ‘Maine’s third delay’ [12], and can mean that the ‘decision to delivery interval’ may be measured in days rather than minutes in some areas.

## Post-caesarean section fistula: traumatic or ischaemic?

Incontrovertibly, a woman neglected in obstructed labour for several days, ultimately delivering a stillborn infant vaginally, then surviving to develop a vesicovaginal fistula (VVF), should be described as having a ‘true obstetric’ or ischaemic fistula. In contrast, a woman identified as being in obstructed labour who is transferred expeditiously to a healthcare facility for timely caesarean section, delivering a live infant, who then develops a vesicocervical fistula, would be reasonably described as having an iatrogenic injury. But what of those women who are transferred only after considerable delay, or who undergo caesarean section for a dead baby and then develop a VVF? Or those who undergo caesarean section but then develop an urethrovaginal fistula or a midvaginal VVF or a ureterovaginal fistula? Should these lesions be looked on as ‘obstetric’, iatrogenic, or a combination?

In Tanzania it is reported that 85 % of patients developing fistula now deliver in hospital, usually by caesarean section, with an increasing rate of vesicocervical and vesicouterine (probably iatrogenic) fistulae. Onsrud et al.*,* in the Democratic Republic of Congo (DRC), have also reported a higher rate of uterine or cervical involvement, with less surrounding fibrosis, and less apparent treatment delay in post-caesarean section fistulae [32]. They consider these to be a distinct clinical (iatrogenic) entity and highlighted the training issues discussed above. Loposso et al. similarly highlighted the risks of fistula in DRC despite the availability of caesarean section, but attributed this to the duration of obstructed labour prior to hospitalisation, and the delay in achieving caesarean delivery [33]. The various colleagues with whom the author has discussed this issue (see Acknowledgments), all with much greater experience of working in different low-income and middle-income countries in Africa and Asia, have given a range of views on the point. Hence, it is likely in different circumstances fistulae following caesarean section may have varying pathophysiology, some being wholly ischaemic, some wholly traumatic, but most with a combination of mechanisms.

Similar arguments may be framed around post-caesarean section ureterovaginal fistulae. Although these are commonly looked on as being exclusively iatrogenic in aetiology, operating on, or close to, the ureter within an ischaemic field defect inevitably puts it at greater risk, and means that ureterovaginal fistula developing after caesarean section for obstructed labour is likely to be at least in part ischaemic in nature.

With the increased use of caesarean section, and consequent increase in ‘repeat’ procedures, the rate of placenta praevia and associated abnormal placental implantation will continue to rise [34–36]. Given the technical difficulties of surgery in these circumstances, we might also anticipate a progressive increase in the rate of fistula following caesarean section. Although the association has been reported [37], a trend is not yet evident, and in the author’s own series from the UK, fistula following caesarean section or trial of attempted vaginal birth after caesarean have consistently represented 6 – 8 % of cases referred over the last three decades [38].

## Accepted views on aetiology of urogenital fistula in high-income countries

Chassar Moir reviewed the position of VVF in Britain in 1973 [39], and Lee et al. similarly in the USA in 1988 [40]. The largest recent UK case series reflects supraregional tertiary care practices, reported by Hilton in 2012 (348 cases) [38]. In the latter series, two-thirds of cases were of surgical aetiology, with the remainder evenly distributed between ‘obstetric’, radiation-induced and miscellaneous or traumatic causes. Although any surgery carried out in the pelvis can be complicated by the development of fistula (Hillary et al., submitted for publication) [38], reports from high-income countries have shown that 70 % of surgical fistulae and 50 % of all lower urinary tract fistulae follow hysterectomy [41]. Less than 20 % of fistulae in high-income countries follow childbirth, and most of these follow obstetric interventions (caesarean section, caesarean hysterectomy, instrumental vaginal delivery, and symphysiotomy), rather than obstructed labour, and should be looked on as being iatrogenic in nature.

## Recent trends in iatrogenic/surgical fistula

In a study using national Hospital Episode Statistics (HES) in England, although overall the numbers were small in global terms, Cromwell and Hilton found a 37 % increase in the number of repair or diversion operations carried out for lower urinary tract fistulae between 2002 and 2009. Considering only primary fistula repair procedures, they found a 68 % increase over the same period (from 62 primary repairs in 2002 to 104 in 2009) [42]. In a further study the same authors found that the number of hysterectomies undertaken in England over a similar period for selected benign and malignant indications fell by 12 % (from 43,014 in 2000 to 37,923 in 2008) [41]. They went on to examine the risk of VVF and urethrovaginal fistula following hysterectomy in the National Health Service in England, and found this to vary both with the indication for surgery and type of procedure undertaken, but overall increased by almost 50 % over the course of the decade studied, from 0.15 % (1 in 681 hysterectomies) in 2000 – 2002 to 0.22 % (1 in 465 hysterectomies) in 2006 – 2008 [41]. In a later study of ureteric injury and ureterovaginal fistula associated with hysterectomy the same group found the risk more than doubling over time, from 0.29 % (1 in 345 hysterectomies) in 2001 – 2005 to 0.66 % (1 in 142 hysterectomies) in 2006 – 2010 [43]. A similar trend over time in post-hysterectomy VVF has recently been reported in the USA [44].

## Factors behind recent trends seen in iatrogenic fistulae in high-income countries

There are several possible factors that might contribute to the findings in relation to iatrogenic (post-surgical) fistulae in the UK. The reduction in the number of hysterectomy procedures undertaken may lead to a relative increase in the number of more complex procedures being carried out, with a higher risk of operative morbidity. However, Hilton and Cromwell did not find the rate of fistula to be significantly higher in women undergoing an abdominal hysterectomy for fibroids or endometriosis than in those with menstrual problems, making this explanation unlikely [41]. The reduction in gynaecological surgical activity overall, with more widespread use of non-surgical treatments, particularly for menorrhagia, the reduced length of trainees’ working week consequent upon the European Working Time Directive, and reduced years in training, all mean that trainees, in the UK at least, obtain less surgical experience now than previously, and may therefore embark on independent practice less prepared than a decade ago.

## Future research

How might one obtain further information to confirm or refute these impressions? Throughout history the establishment of causality in medicine has proved elusive [45, 46], and the confirmation of secular trends in causality presents even greater challenges. Difficult as these issues have been to establish in the context of infectious diseases [47], they take on a completely different magnitude with a problem as complex as obstetric fistula, given its physical, biosocial, cultural, economic and geopolitical contributors. Data collection is clearly important, but even such powerful tools as the Demographic and Health Surveys Program can provide only a small proportion of the information needed to fully investigate these issues [48, 49]. Almost all of the United Nations Millennium Development Goals potentially impact on the causation and management of fistula [50, 51], and yet there remain substantial gaps in our achievement of these goals [52]. Given a worldwide fistula prevalence of up to 3,500,000 [7], and an incidence of up to 1 in 800 births [16], there is clearly much to be done before we can claim to be effectively managing and preventing this condition.

## Conclusions

There is a growing perception that with more widespread understanding of the causes of obstetric fistulae in low-income and middle-income countries, and *increasing* rates of caesarean section, there is an *increase* in VVF and ureterovaginal fistulae that may be at least in part iatrogenic in nature. There is evidence to support the possibility of an *increase* in the incidence of iatrogenic (post-hysterectomy) fistulae in high-income countries, which coincides with a *decrease* in the number of hysterectomies carried out. In the first case this may reflect the amount of training, supervision and support provided to trainees; in the second it may reflect the amount of surgical experience accrued in training and workload in independent practice. In both situations it behoves governments and educational authorities to invest in training and workforce planning, and to facilitate audit and operational research, so that an appropriately trained and supervised medical workforce is maintained in the correct working environment. Alternatively, so that the right people do the right thing at the right time and in the right place. Only then can our surgical evolution in this area undergo the quantum change required to move from its present retrogressive position to become progressive.

*Plus ça change, plus ça peut s’améliorer* – after J.-B.A. Karr (and G.W. Leibniz)

## References

[1] Merriam-Webster Inc (2015) Merriam-Webster medical dictionary. Available from: http://www.merriam-webster.com/browse/medical/a.htm. Accessed 10 Dec 2015

[2] Basinger Maggenti MA, Maggenti AR (2005) Online dictionary of invertebrate zoology. University of Nebraska - Lincoln (digitalcommons.unl.edu). Available from: http://digitalcommons.unl.edu/onlinedictinvertzoology/2. Accessed 10 Dec 2015

[3] Darwin C (1859). On the origin of species by means of natural selection, or the preservation of favoured races in the struggle for life.

[4] Cope ED (1858). On the origin of genera. Proc Acad Natl Sci Phila.

[5] Cope ED (1885). On the evolution of the vertebrata, progressive and retrogressive. Am Nat.

[6] Huxley TH (1894). The struggle for existence in human society. Evolution and ethics, volume IX of Huxley’s Collected Essays.

[7] Abrams P, de Ridder D, de Vries C, Elneil S, Esegbona G, Mouread S (2012). ICUD-SIU 1st international consultation on obstetric fistula in the developing world.

[8] de Ridder D, Hilton P, Mourad S, Pickard RS, Rovner ES, Stanford E, Abrams P, Cardozo LD, Khoury S, Wein A (2013). Fistulae. Incontinence: 5th International Consultation on Incontinence.

[9] Safan A, Shaker H, Abdelaal A, Mourad MS, Albaz M (2009). Fibrin glue versus martius flap interpositioning in the repair of complicated obstetric vesicovaginal fistula. A prospective multi-institution randomized trial. Neurourol Urodyn.

[10] Shaker H, Saafan A, Yassin M, Idrissa A, Mourad MS (2011). Obstetric vesico-vaginal fistula repair: should we trim the fistula edges? A randomized prospective study. Neurourol Urodyn.

[11] Tomlinson AJ, Thornton JG (1998). A randomised controlled trial of antibiotic prophylaxis for vesico-vaginal fistula repair. BJOG.

[12] Thaddeus S, Maine D (1994). Too far to walk: maternal mortality in context. Soc Sci Med.

[13] Muleta M, Fantahun M, Tafesse B, Hamlin EC, Kennedy RC (2007). Obstetric fistula in rural Ethiopia. East Afr Med J.

[14] Kalilani-Phiri LV, Umar E, Lazaro D, Lunguzi J, Chilungo A (2010). Prevalence of obstetric fistula in Malawi. Int J Gynaecol Obstet.

[15] Walz NK, Faroqul M, Begum A, Sultana N, Sarker S, Faisel AJ (2003). Situation analysis of obstetric fistula in Bangladesh.

[16] Vangeenderhuysen C, Prual A, Ould el Joud D (2001). Obstetric fistulae: incidence estimates for sub-Saharan Africa. Int J Gynaecol Obstet.

[17] Goh JT, Browning A, Berhan B, Chang A (2008). Predicting the risk of failure of closure of obstetric fistula and residual urinary incontinence using a classification system. Int Urogynecol J Pelvic Floor Dysfunct.

[18] Goh J (2004). A new classification for female genital tract fistula. Aust N Z J Obstet Gynaecol.

[19] Raassen TJ, Ngongo CJ, Mahendeka MM (2014). Iatrogenic genitourinary fistula: an 18-year retrospective review of 805 injuries. Int Urogynecol J.

[20] Hilton P, Ward A (1998). Epidemiological and surgical aspects of urogenital fistulae: a review of 25 years experience in south-east Nigeria. Int Urogynecol J Pelvic Floor Dysfunct.

[21] World Health Organization (2015) WHO statement on caesarean section rates. Available from: http://apps.who.int/iris/bitstream/10665/161442/1/WHO_RHR_15.02_eng.pdf. Accessed 10 Dec 2015

[22] WHO, UNFPA, UNICEF (2009). Averting maternal death and disability (AMDD). Monitoring emergency obstetric care: a handbook.

[23] Cavallaro FL, Cresswell JA, Franca GV, Victora CG, Barros AJ, Ronsmans C (2013). Trends in caesarean delivery by country and wealth quintile: cross-sectional surveys in southern Asia and sub-Saharan Africa. Bull World Health Organ.

[24] Duong TH, Patterson TM (2014). Lower urinary tract injuries during hysterectomy in women with a history of two or more Cesarean deliveries: a secondary analysis. Int Urogynecol J.

[25] Oliphant SS, Bochenska K, Tolge ME, Catov JM, Zyczynski HM (2014). Maternal lower urinary tract injury at the time of Cesarean delivery. Int Urogynecol J.

[26] Shah A, Fawole B, M’Imunya JM, Amokrane F, Nafiou I, Wolomby JJ (2009). Cesarean delivery outcomes from the WHO global survey on maternal and perinatal health in Africa. Int J Gynaecol Obstet.

[27] Mullan F, Frehywot S (2007). Non-physician clinicians in 47 sub-Saharan African countries. Lancet.

[28] Kruk ME, Pereira C, Vaz F, Bergstrom S, Galea S (2007). Economic evaluation of surgically trained assistant medical officers in performing major obstetric surgery in Mozambique. BJOG.

[29] McCord C, Mbaruku G, Pereira C, Nzabuhakwa C, Bergstrom S (2009). The quality of emergency obstetrical surgery by assistant medical officers in Tanzanian district hospitals. Health Aff (Millwood).

[30] Chilopora G, Pereira C, Kamwendo F, Chimbiri A, Malunga E, Bergstrom S (2007). Postoperative outcome of caesarean sections and other major emergency obstetric surgery by clinical officers and medical officers in Malawi. Hum Resour Health.

[31] Wilson A, Lissauer D, Thangaratinam S, Khan KS, MacArthur C, Coomarasamy A (2011). A comparison of clinical officers with medical doctors on outcomes of caesarean section in the developing world: meta-analysis of controlled studies. BMJ.

[32] Onsrud M, Sjoveian S, Mukwege D (2011). Cesarean delivery-related fistulae in the Democratic Republic of Congo. Int J Gynaecol Obstet.

[33] Loposso MN, Ndundu J, De Win G, Ost D, Punga AM, De Ridder D (2015). Obstetric fistula in a district hospital in DR Congo: fistula still occur despite access to caesarean section. Neurourol Urodyn.

[34] Johnston TA, Paterson-Brown S, Guidelines Committee of the RCOG (2011) Placenta praevia, placenta praevia accreta and vasa praevia: diagnosis and management. Green-top Guideline no. 27. Available from: https://www.rcog.org.uk/globalassets/documents/guidelines/gtg_27.pdf. Accessed 10 Dec 2015

[35] Silver RM, Landon MB, Rouse DJ, Leveno KJ, Spong CY, Thom EA (2006). Maternal morbidity associated with multiple repeat Cesarean deliveries. Obstet Gynecol.

[36] Zuarez-Easton S, Shalev E, Salim R (2015). Trend in major neonatal and maternal morbidities accompanying the rise in the cesarean delivery rate. Sci Rep.

[37] Mann S, Dahiya P, Mann A, Madan S (2013). Uterovesicovaginal fistula following placenta percreta: case review and management options. Urogynaecologia.

[38] Hilton P (2012). Urogenital fistula in the UK – a personal case series managed over 25 years. BJU Int.

[39] Chassar Moir J (1973). Vesico-vaginal fistulae as seen in Britain. BJOG.

[40] Lee RA, Symmonds RE, Williams TJ (1988). Current status of genitourinary fistula. Obstet Gynecol.

[41] Hilton P, Cromwell D (2012). The risk of vesicovaginal and urethrovaginal fistula after hysterectomy performed in the English National Health Service – a retrospective cohort study examining patterns of care between 2000 and 2008. BJOG.

[42] Cromwell D, Hilton P (2013). Retrospective cohort study on patterns of care and outcomes of surgical treatment for lower urinary-genital tract fistula among English National Health Service hospitals between 2000 and 2009. BJU Int.

[43] Kiran A, Hilton P, Cromwell DA (2015) The risk of ureteric injury associated with hysterectomy: a 10-year retrospective cohort study. BJOG. doi:10.1111/471-0528.13576. Accessed 10 Dec 2015 10.1111/1471-0528.13576 26281794

[44] Adam RA, Graves A, Ni S, McPheeters M (2015) Time trends in post-hysterectomy vesicovaginal fistula and lower urinary tract injury. J Minim Invasive Gynecol 22:S7–S8 (abstract)

[45] Evans AS (1978). Causation and disease: a chronological journey. The Thomas Parran lecture. Am J Epidemiol.

[46] Feezer LW (1921). Theories concerning the casusation of disease. Am J Public Health.

[47] Evans AS (1976). Causation and disease: the Henle-Koch postulates revisited. Yale J Biol Med.

[48] Demographic Health Surveys. The DHS program. Available from: http://dhsprogram.com. Accessed 10 Dec 2015

[49] Johnson K, Peterman A (2008). Incontinence data from the Demographic and Health Surveys: comparative analysis of a proxy measurement of vaginal fistula and recommendations for future population-based data collection. DHS Analytical Studies.

[50] United Nations (2006) UN millennium project. Available from: http://www.unmillenniumproject.org/index.htm. Accessed 10 Dec 2015

[51] United Nations General Assembly. United Nations Millennium Declaration 2000: A/RES/55/2

[52] United Nations (2015). The millennium development goals report.

